# Intermetallic compounds in heterogeneous catalysis

**DOI:** 10.1080/14686996.2020.1831272

**Published:** 2020-11-02

**Authors:** Marc Armbrüster, Yuri Grin

**Affiliations:** a Faculty of Natural Sciences, Institute of Chemistry, Materials for Innovative Energy Concepts, Chemnitz University of Technology, Chemnitz, Germany; b Chemical Metals Science Department, Max-Planck-Institut für Chemische Physik fester Stoffe, Dresden, Germany

Chemical transformations by heterogeneous catalysis enabled fundamental research and chemical industry to support the technical development and stabilize the prosperity of the human community for – at least – the last hundred years. The Haber-Bosch process providing vast amounts of cheap ammonia and its importance for a wide spectrum of industrial branches might serve as a vivid example. Complex changes in the human society and the relation of the latter to nature, especially the protection of the environment, require the optimization of known and development of new chemical processes yielding the targeted products with minimal possible damage to the pristine nature.

A deep understanding of the ongoing processes on the interface between the participating phases in heterogeneous catalysis results in optimized materials and enables the development of urgently needed new processes, e.g. in the context of renewable energy storage. In the simplest case, heterogeneous catalysis involves only three general steps – adsorption of the reactants, reaction of the adsorbed reactants and desorption of the products – all happening on the active sites located on the accessible surface of the catalysts. One of the key issues in understanding heterogeneous catalysis is, therefore, the question of the formation and atomic decoration of the active sites as well as the role of their electronic structure. The nucleus of active sites is often a transition metal. In the traditional catalytic processes, such active sites may be located on a catalytically non-active support or are indeed a part of the support, most commonly an oxide. The so-obtained catalyst is obviously very complex, one may take as an example the classical Cu/zinc oxide/aluminum oxide catalyst for the methanol synthesis. The understanding of the catalytic mechanism on an atomic-molecular level in such systems is strongly hindered simply by the large number of reactions and processes possible. The use of unsupported catalysts based on metallic alloys reduces the number of possible elementary processes on the surface. The large number of different atomic configurations on the surface due to the presence of extended solid solutions in alloys still does not allow for an easy development of more transparent and provable reaction mechanisms – especially when severe segregation of one of the components is occurring. A way out of this dilemma may be achieved employing intermetallic compounds as unsupported catalysts. Their ordered crystal structures are caused by the special bonding feature – a coexistence of two- and multi-center atomic interactions – and contain atomic arrangements markedly deviating from the closest packing patterns characteristic for alloys. For the same bonding reasons, the electronic structure of intermetallic compounds also differs strongly from the ones of elemental metals or their alloys. Thus, the use of intermetallic compounds as catalysts allows to influence both – geometrical and electronic – factors controlling the general steps of heterogeneous catalysis mentioned above. Further typical but not general advantages of the intermetallic compounds are their high thermodynamic stability (e.g. intermetallic phases of boron and transition metals are stable far above thousand degrees centigrade), chemical resistivity, mechanic stability, etc., which may play a key role considering the practical manufacturing of heterogeneous catalysts.

All this gives a rough idea about the huge research field of heterogeneous catalysis on intermetallic compounds. The special issue of Science and Technology of Advanced Materials offers to the reader current results obtained by research groups around the world on this topic.

This Special Issue was initiated together with our honorable colleague – Professor An Pang Tsai from the Tohoku University in Sendai, Japan. Much to our regret, An Pang Tsai passed away during the work on this project. The Editors of the Special Issue and the authors of the included publications dedicate this work to the memory of An Pang Tsai.

